# Bioactive protein hydrolysate from *Sesamum indicum* L. residue as a novel fat substitute by protease: production optimization and application in low-fat yogurt production

**DOI:** 10.1186/s12934-025-02748-3

**Published:** 2025-05-27

**Authors:** Samia A. Ahmed, Mohamed A. A. Abdella, Osama A. Ibrahim

**Affiliations:** 1 Chemistry of Natural and Microbial Products Department, Pharmaceutical and Drug Industries Research Institute, National Research Centre, Giza, Egypt; 2 Dairy Science Department, Industries and Nutrition Research Institute, National Research Centre, Giza, Egypt

**Keywords:** Fat-substitutes, Protease, Protein-hydrolysate, Sesame cake, Low-fat yogurt

## Abstract

**Background:**

Agricultural and industrial residues are renewable biomass sources present in large quantities causing pollution. Therefore, transforming these residues to eco-friendly products such as enzymes and bioactive materials reduces their quantity and impact on the environment, in addition to reducing the production costs.

**Results:**

Sesame cake is a by-product of the production of Sesame seed oil and is high in protein. The yield of Sesame cake protein hydrolysis (SH) improved by 4.2-fold through the optimization of conditions using *Bacillus thuringiensis* strain-MA8 protease via the Box-Behnken design (BBd). The average diameter of the particle size of SH was 677.10 nm. The application of SH (1–3%) in the production of low-fat yogurt (LSH) exhibited a fermentation time similar to that enriched with skim milk powder (LSMP). The total solids and protein levels in LSH-yogurt exceeded those in full fat yogurt (FFY). In addition, the acidity and overall acceptability ratings of LSH-yogurt were similar to FFY throughout the 15-day storage at 5 °C, without displaying any defects. Furthermore, the total essential amino acids (TEAA), total amino acids (TAA), and TEAA/TAA ratio of LSH (2%)-yogurt were approximately similar to FFY. Incorporating SH (2%) improved the chemical score of certain amino acids in LSH-yogurt. The hardness of LSH-yogurt exceeded that of FFY. Additionally, the springiness, gumminess, and cohesiveness of LSH-yogurt were similar to those of LSMP.

**Conclusions:**

Protein hydrolysate from Sesame cake is a new fat substitute for low-fat yogurt production without displaying any defects as well as reducing the risks associated with high-fat consumption and global obesity.

**Supplementary Information:**

The online version contains supplementary material available at 10.1186/s12934-025-02748-3.

## Highlights

Eco-friendly way to convert Sesame cake residues to bioactive protein hydrolysate (SH).

Box-Behnken optimization promote hydrolysate yield by 4.2-fold.

Low-fat yogurt supplemented with SH takes a shorter fermentation time.

SH-yogurt acceptance scores were close to full fat yogurt (FFY) with no defects.

Total solids, protein contents, and amino acids of SH-yogurt were higher than FFY.

## Introduction

The world’s population is increasing every year, leading to an increased demand for food supplies and proteins [1].

Yogurt is a fermented dairy product and is one of the most important dairy products consumed worldwide due to its high nutritional value, functional properties, variety of flavors and forms, and reasonable price [2, 3].

As a result of the risks associated with excessive fat intake and obesity worldwide, there is an increasing consumption of low-fat dairy items. However, the elimination of fat from yogurt raises certain issues regarding decreased viscosity, poor (body and texture), sensory quality, and synergy [4]. Consequently, it is essential to find methods to enhance the characteristics of yogurt. One of these techniques involves utilizing fat substitutes. Some fats in foods can be replaced with certain ingredients that provide some of the same properties as fats. These types of fat substitutes can be proteins, carbohydrates, or lipids [5]. The fat-mimicking property can be enhanced by a combination of animal or plant protein.

From economic and environmental perspectives, the conversion of biological waste into high value-added compounds has attracted considerable attention from scientific and commercial entities [6]. Recycling food processing residue is an important industrial process to obtain high value-added protein sources [7]. Because of their rich nutritional content, these residues are viewed as raw materials for creating and developing other products rather than being seen as waste [8]. To lower the ultimate expense and ensure future management of food resources, protein hydrolysates are frequently created from leftover proteins and by-products of the food sector [8].

Protein hydrolysates are complex mixtures obtained from various protein sources using several techniques [9]. Protein hydrolysates are widely used as functional ingredients, nutritional supplements, flavor enhancers in food, coffee whiteners, personal care cosmetics, and various other uses [10]. Lately, the unique functional characteristics and sustainability of plant-derived proteins have become highly valued by consumers and the food industry. Population growth, ethical considerations, safety concerns, and the need for cleaner, healthier food options are also fueling the consumption of plant-based proteins [11]. Moreover, they are easy to digest, non-harmful, versatile, rich in nutrients, the isolation process is less expensive, and have no side effects [1, 12]. In addition, the inclusion of hydrolyzed plant protein has demonstrated enhancements in both the quality and taste of fermented foods [3].

Sesame (*Sesamum indicum* L.) is one of the first oil crops used by humans. Sesame has been grown for centuries, particularly in Africa and Asia [10]. In the Sesame oil industry, Sesame oil is extracted and Sesame cake (SC) is a by-product after oil extraction, containing approximately 40% protein, 22.7% crude fiber, elevated calcium levels, minerals, vitamins, and trace elements [13, 14]. SC, a by-product of oil crops, has low exploitation value and is prone to causing environmental problems. Developing proteins in stem cells into hydrolysates not only solves environmental pollution problems, but also creates more valuable and lower-cost food additives [15]. Sesame cake provides health benefits when consumed. Specifically, it is rich in protein, and contains mostly albumins and globulins, as well as glutelins and prolamins sub-fractions. These sub-fractions can enhance the nutritional value of food products due to their composition of amino acids, specifically, methionine and cysteine. Therefore, there is great interest in using sesame cake protein hydrolysate in food products to enhance their nutritional value [16].

Two different methods are used to achieve protein hydrolysate: chemical (acidic or alkaline) and enzymatic (proteases) [17, 18]. Chemical hydrolysis occurs in a non-specific way, leading to the possible destruction of products and amino acids with high ash content. Additionally, certain chemicals increase toxicity, and require harsh conditions for hydrolysis [8]. The enzymatic method is the most favorite method due to its low cost, large-scale commercial availability, and better control of the resulting hydrolysate and hence its properties [7, 18]. Moreover, it demands gentle reaction conditions, produces minimal byproducts, promotes high yield, and ensures product quality [11, 19]. A successful method for acquiring natural food additives is via enzymatic hydrolysis, which also aims to create unique food products that enhance health [20]. In order to create a hydrolysate suitable for use as a food additive, it should be made with a food-grade enzyme preparation or a commercially available enzyme that is cost-effective for the producer [8]. The majority of commonly utilized commercial enzyme products are derived from animal and microbial origins. The microbial enzymatic hydrolysis process offers benefits such as high analytical specificity, gentle reaction conditions, elevated product purity, and reduced energy needs [21].

The output of protein hydrolysate can be enhanced since it is influenced by various factors including enzyme type, hydrolysis duration, pH, substrate concentration, enzyme/substrate ratio, and temperature [8, 19]. Optimizing hydrolysis conditions is essential to produce the highest yield [22]. Response surface methodology (RSM) is an effective approach for examining intricate processes, which has been utilized to enhance processing parameters [22, 23].

However, whether the application of Sesame cake protein hydrolysate (SH) in dairy products has a potential impact on dairy processing and product quality is unsolved in the present time, which might be an important issue to the yogurt producers. Based on this consideration, the optimization of SH production by bacterial protease was carried out using BBd. Also, the current study assessed the effect of SH (as fat substitutes) on the set-style low-fat yogurt samples using indices like acid production, textural, chemical, sensorial, and nutritional properties as evaluation criteria. Interestingly, this is the first study on the use of SH as a fat substitute in the production of low-fat yogurt.

## Materials and methods

### Raw material and chemicals

Fresh full fat and skim buffalo milk were sourced from the Faculty of Agriculture, Cairo University, Egypt. Skim milk powder (SMP) was sourced from BIELMLEK Spoldzielnia mleczrska, Poland. All chemicals used in this study were of analytical grade.

### Collection and preparation of waste materials (residue) and by-products (WMBP)

WMBP comprising corn cob (CC), lupine seeds (LS), Sesame cake (SC), soybean (SB), tea waste (TW), and wheat straw (WS) were locally collected, washed with water, dried, grinded by blender, sifted, and put in an airtight vessel.

### Protease activity assessment

Protease enzyme was produced by *Bacillus thuringiensis* strain-MA8 through submerged fermentation as formerly described by Abdella et al. [24]. Protease activity was evaluated following the adapted technique of Shafique et al. [25] using casein as the substrate. To initiate the interaction, 0.5 mL of crude enzyme solution was combined with 0.5 mL of 1% casein dissolved in 0.1 M sodium phosphate buffer (pH 7.0) and the mixture was incubated in a water bath for 30 min at 40 °C. To terminate the reaction, 1 mL of 10% trichloroacetic acid (TCA) was added to the mixture, allowed to sit for 10 min at room temperature, and subsequently centrifuged at 10,000 ×*g* for 15 min. The protein hydrolysate in the supernatant was estimated according to Lowry et al. [26], using bovine serum albumin as the standard. All experiments were conducted in triplicate and the results were expressed as the mean ± standard deviation. One unit (U) of enzymatic activity was defined as the quantity of enzyme that generates one µg of amino acid (as tyrosine) per min under testing conditions.

### Degradation of WMBP by protease enzyme and protein hydrolysate estimation

A certain amount (0.1 g) of the WMBP (CC, LS, SC, SB, TW, and WS) was mixed with 0.25 mL (60 U) of protease enzyme and 0.5 mL of sodium phosphate buffer (pH 7.0) in a shaker (150 rpm) at 40 °C for varying incubation times (3, 6, 12, and 24 h). Subsequently, the mixture was heated at 95 °C for 15 min to deactivate the enzyme, followed by centrifugation at 10,000 ×*g* and 4 °C for 15 min [8]. The supernatant was utilized to determine protein hydrolysate (mg/mL/g WMBP) using the method of Lowry et al. [26].

### Response surface methodology using BBd for optimizing protein hydrolysate conditions

BBd was applied to obtain optimal values for several key variables affecting protein hydrolysate yield by *B. thuringiensis* strain-MA8 protease [27]. In the current study, three levels identified as (-1, 0, + 1) were evaluated for the three chosen variables including: enzyme units, temperature, and pH as displayed in Table 1. According to the BBd, 15 experimental trials were created from the combinations of variables, and the statistical results (predicted response) were analyzed using a second-order (quadratic) polynomial model represented by the following equation:

**Table 1:** Plackett-Burman (PB) design to optimize variables influencing protein hydrolysate production (yield) by *Bacillus thuringiensis* strain-MA8 protease

Trial	A: Enzyme units	B: Temperature	C: pH	Protein hydrolysate	Predicted response
	U	°C	-	mg/mL/g	mg/mL/g
1	(0)150	(+ 1)50	(+ 1)8	240	250.90
2	(-1)60	(-1)30	(0)7	123.2	133.40
3	(+ 1)240	(+ 1)50	(0)7	324.8	314.60
4	(0)150	(0)40	(0)7	172	175.27
5	(0)150	(0)40	(0)7	172.1	175.27
6	(+ 1)240	(-1)30	(0)7	277.6	284.80
7	(+ 1)240	(0)40	(-1)6	328	331.70
8	(0)150	(+ 1)50	(-1)6	358.4	364.90
9	(-1)60	(0)40	(+ 1)8	152	148.30
10	(-1)60	(0)40	(-1)6	196	196.70
11	(0)150	(0)40	(0)7	172	175.27
12	(+ 1)240	(0)40	(+ 1)8	319.2	318.50
13	(0)150	(-1)30	(+ 1)8	312	305.50
14	(-1)60	(+ 1)50	(0)7	168	160.80
15	(0)150	(-1)30	(-1)6	264	253.10

$$Y\, = \,{\beta _0}\, + \,\Sigma {\beta _i}{X_i}\, + \,\Sigma {\beta _{ii}}{X_i}^2\, + \,\Sigma {\beta _{ij}}{X_i}{X_j}$$

Where: Y represents the predicted response, β_0_ denotes the intercept term, β_i_ signifies the linear coefficient, β_ii,_ represents the quadratic coefficient, β_ij,_ signifies the interaction coefficient, while X_i_, and X_j,_ are the independent variables.

### Particle size of Sesame cake protein hydrolsate (SH)

The particle size distribution of SH resulted from *Bacillus thuringiensis* strain-MA8 protease treatment was measured using NICOMP 380 ZLS, a Dynamic light scattering (DLS) device (PSS, Santa Barbara, CA, USA).

### Preparation of yogurt

Yogurt was prepared using fresh skim buffalo milk as per Robinson and Tamime [28]. The yogurt milk was split into 5 portions, the first portion served as a control (low-fat yogurt fortified with 2% skim milk powder, LSMP), the second portion is low-fat yogurt fortified with 2% Sesame cake protein powder (LSP), while the remaining three portions were fortified with 1, 2, and 3% of Sesame cake protein hydrolysate (SH) utilizing *Bacillus thuringiensis* strain-MA8 protease. Control sample of full fat yogurt (FFY) was made from full fat buffalo milk without adding any protein (F).

Yogurt milk was heated at 85 °C for 5 min and subsequently cooled to 42 °C, after which it was inoculated with 2% of a yogurt starter culture containing *Streptococcus thermophiles* CH-1 (Chr. Hansens’s Lab., Denmark) and *Lactobacillus bulgaricus* Lb-DRI-VAC (Northern Regional Research Lab. Illinois, USA) at a ratio of 1:1 which contained about 2.5 × 10^10^ cfu/mL. All inoculated yogurt milk treatments were placed into appropriate cups and incubated at 42 °C until fully coagulated. Then, the yogurt containers were cooled and stored at 5 °C prior to evaluation after 0, 7, and 14 days. Two replications of the whole experiment and parameters analyses were performed.

### Determination of fermentation time

The pH readings throughout the yogurt fermentation process were recorded with a digital pH-meter featuring glass electrode, Ingold, Knick, Germany. The fermentation time was established as the period needed for the milk coagulum to reach a pH level of 4.6.

### Chemical characterization of yogurt

The yogurt samples were subjected to chemical analysis in accordance with the Association of Official Analytical Chemistry [29] for total solids, fat content, ash, total protein, and acidity as a lactic acid.

### Yogurt rheological analysis

Texture profile analyses (TPA) of yogurt samples were performed with a Universal Testing Machine (Co metech, B type, Taiwan) equipped with a 25-mm-diameter perplex conical-shaped probe, and the resulting plot of force (N) against time (s) was documented. TPA parameters were established based on the definition provided by the International Dairy Federation [30] from the resulting force-time curve for textural attributes. The hardness, chewiness, cohesiveness, gumminess, and springiness were calculated as follows: Hardness (N) = Maximum force of the 1st compression → {1}.

Cohesiveness area = Area under the 2nd compression / Area under the 1st compression (B/A) → {2}.

Springiness (mm) = Length 2nd compression / Length 1st compression (L2/L1) → {3}.

Gumminess (N) = Hardness × Cohesiveness → {4}.

Chewiness (N/mm) = Gumminess × Springiness → {5}.

### Yogurt sensory evaluation

The sensorial characteristics of yogurt during storage at 5 °C after 1, 7, and 14 days were evaluated by means of regular 10 descriptive panelists from the researchers’ staff of Dairy Science Department, National Research Centre (Cairo, Egypt) according to Pappas et al. [31]. The yogurt samples (100 ml cups) were evaluated with a top score of 50 points for flavor including taste, mouth feels, and odor (0-bad, 50-very good), 40 points for body and texture including texture, consistency, and viscosity (0–weak, 40–gel-like), 10 points for the yogurt appearance including color and shape (0–shrunken, 10–a typical appearance), and overall acceptability describes all tested sensorial attributes of yogurt samples.

### Yogurt amino acids profile

The compositions of amino acids in yogurt samples were analyzed using the high-performance liquid chromatography (HPLC)-Pico-Tag method [32–34]. Phenylisothiocyanate (PITC, also known as Edman’s reagent) was employed for pre-column derivatization, and reversed-phase gradient elution HPLC was utilized to separate the phenylthiocarbamyl (PTC) derivatives detected through their UV absorbance. The Liquid chromatography apparatus was fitted with a 600 E Multisolvent Delivery System and utilized the subsequent gradient of Pico-Tag solvents A and B (Waters Eluent A and B) at 38 °C, with a flow rate 1 mL/min. A sample of twenty microliter was injected and placed onto the Pico-Tag amino acids column (150 × 3.9 mm, stainless steel) via linear gradient elution. The PTC derivatives are detected using ultraviolet absorption at 254 nm (2489 UV/Vis Detector).

### Chemical score, protein efficiency ratio, and biological value of yogurt

Using the amino acids profile of yogurt, the chemical score (CS) [35], the protein efficiency ratio (PER) [36], and the biological value (BV) [37] were calculated as follows:

CS = (mg of amino acid in 1 g test protein/ mg of amino acid in 1 g reference protein)×100 → {6}.

PER =– 0.468 + 0.454 (leucine)– 0.105 (tyrosine) → {7}.

BV = 49.09 + 10.53 (PER) → {8}

### Data analysis and software

#### Production of protein hydrolysate

The analysis of variance (ANOVA), which includes *F*-test and *P*-value, serves as evidence for the model’s significance and the importance of each term in the quadratic equation. Also, the value of *R*^*2*^ (regression coefficient or determination coefficient) and Adjusted *R*^*2*^ can indicate the strength of the statistical model [38]. Design Expert 13.0 (Stat Ease Inc., Minneapolis, MN, USA) statistical software was used for experimental design, data analysis, and results interpretation.

#### Production of low-fat yogurt

The statistical evaluation of all results was performed by SAS statistical software [39] using the ANOVA method for analysis of variance. The findings were presented as mean ± standard error, and the differences between means were evaluated for significance using Duncan’s multiple ranges at *p* ≤ 0.05.

## Results and discussion

### Degradation of WMBP by protease enzyme and Estimation of protein hydrolysate

**Fig. 1:**
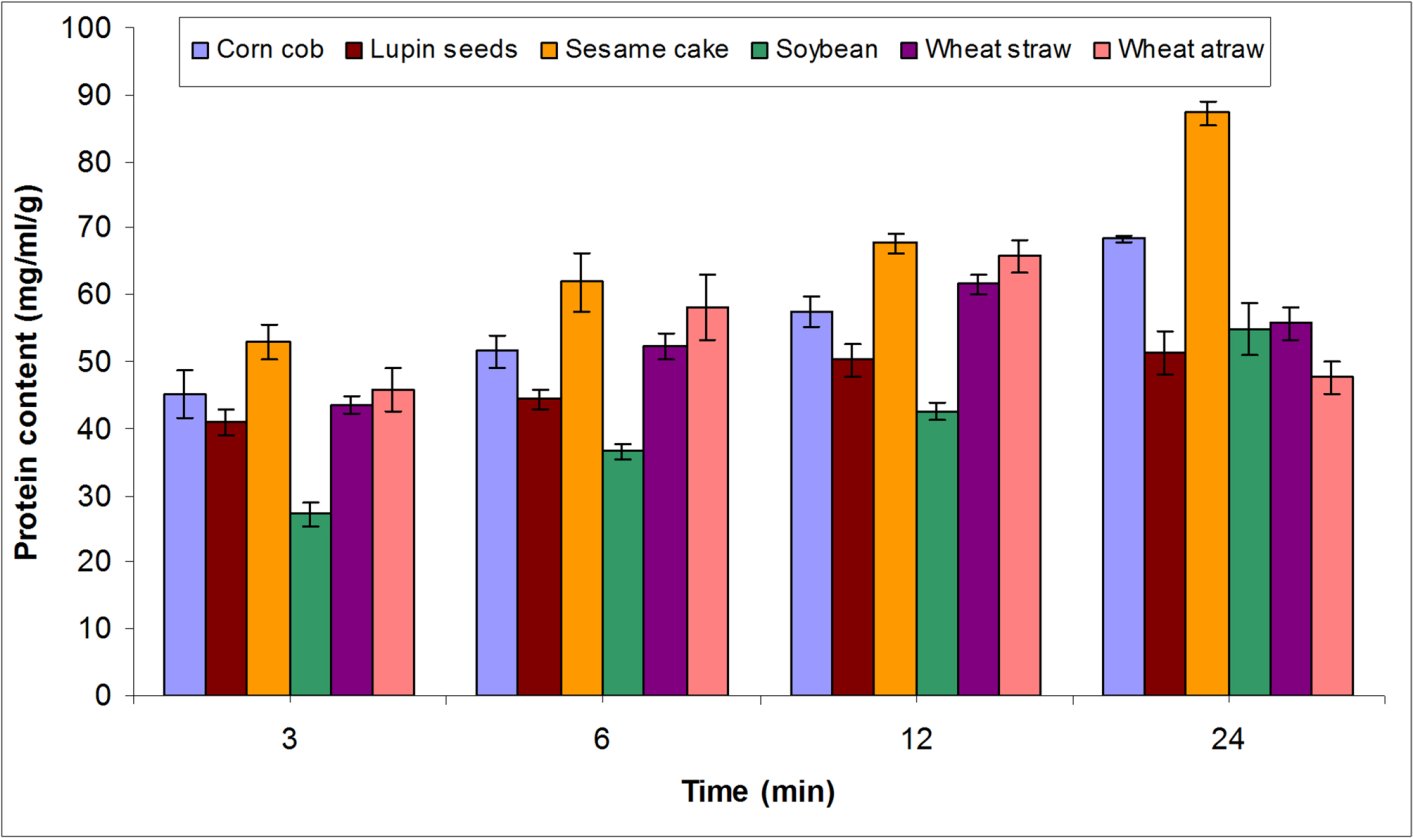
Degradation of various waste materials protein using *B. thuringiensis* strain-MA8 protease

Degradation of WMBP using *B. thuringiensis* strain-MA8 protease to generate protein hydrolysate relies on the type of WMBP and the time required for complete hydrolysis [8]. Based on the findings in Fig. 1, the highest yield of protein hydrolysate was noted after 24 h of SC degradation followed by CC recording 87.2 and 68.4 mg/mL/g. Otherwise, the lowest value of protein hydrolysate (27.1 mg/mL/g) was obtained from SB after 3 h. This result is consistent with the study of De Asis et al. [22], which showed that protein content increased with longer hydrolysis time. Similarly, Ahmed et al. [8] noted that the protein content with *Aspergillus niger* WA 2017 protease changed based on the kind of raw material and duration of hydrolysis.

### Response surface methodology using BBd for optimizing protein hydrolysate conditions

Enzymatic hydrolysis depends on some factors such as enzyme type, enzyme concentration, temperature, pH, time, and nature of substrate [18]. Consequently, optimizing the hydrolysis conditions is crucial to achieve the maximum yield of protein hydrolysate. BBd was performed to obtain the optimal levels of the examined variables required for maximum yield of protein hydrolysate. The combinations between the tested variables (enzyme units, temperature, and pH) introduced 15 trials with their actual and predicted responses that were showed great variations as seen in Table 1. The accomplishment of multiple-regression analysis to the BBd results produced quadratic polynomial model that was appropriate to the following equation:

$$\begin{gathered}Y\, = \,175.27\, + \,76.30A\, + 14.30B\, \hfill \\\,\,\,\,\,\,\,\,\, - \,15.40C\, + \,0.600AB\, + 8.80AC\, \hfill \\\,\,\,\,\,\,\,\,\, - 41.60BC\, + 1.67{A^2}\, + \,46.47\,{B^2}\, + 71.87{C^2} \hfill \\ \end{gathered} $$

Where: Y represents the predicted response (protein hydrolysate mg/mL/g), while A, B, and C denote the values for enzyme units, temperature, and pH.

The ANOVA for BBd was performed to show the importance of the statistical model and every term in the equation. According to the results illustrated in Table 2, the model *F*-value of 63.51 and *P*-value of 0.0001 (*P*-value < 0.05) imply that the model was significant. There is only 0.01% chance that such a large model *F*-value could occur due to noise. Moreover, the *R*^*2*^-value (0.9913) indicates the regression model can provide a good interpretation (99.13%) of the variance in response values (protein hydrolysate). Also, the great accordance between *R*^*2*^-value (0.9913), Adjusted *R*^*2*^-value (0.9757), and Predicted ^*2*^-value (0.9071) proves the fitness of the regression model and the high correlation between the experimental and predicted results [40].

**Table 2:** ANOVA of BBd for optimizing protein hydrolysate production (yield) by *Bacillus thuringiensis* strain-MA8 protease

Source	Sum of Squares	DF	Mean Square	Std. Dev.	F-value	*P*-value	
Model	82807.15	9	9200.79	12.04	63.51	0.0001	Significant
A-Enzyme units	46573.52	1	46573.52	68.03	321.50	< 0.0001	
B-Temperature	1635.92	1	1635.92	7.56	11.29	0.0201	
C-pH	1897.28	1	1897.28	0.7559	13.10	0.0152	
AB	1.44	1	1.44		0.0099	0.9245	
AC	309.76	1	309.76		2.14	0.2035	
BC	6922.24	1	6922.24		47.78	0.0010	
A^2^	10.26	1	10.26		0.0708	0.8008	
B^2^	7972.25	1	7972.25		55.03	0.0007	
C^2^	19070.10	1	19070.10		131.64	< 0.0001	
Residual	724.33	5	144.87				
Lack of Fit	662.24	3	220.75		7.11	0.1258	Non-significant
Pure Error	62.09	2	31.04				
Cor Total	83531.47	14					

*R*^2^**=** 0.9913, Adjusted *R*^2^ = 0.9757, Predicted *R*^2^ = 0.9071, CV = 5.03%, Adequate Precision = 23.56

Std. Dev. (standard deviation), DF (degree of freedom), Significant (*P* < 0.05), Non-significant (*P* > 0.05)

Further, the lack of fit *F*-value (7.11) and *P*-value (0.1258) imply the lack of fit is not significant relative to the pure error. Non-significant lack of fit is good to confirm the adequacy of the regression model that was fitted to the predict response. On the other side, the reliability of the observed data was achieved via the coefficient of variation (CV) value which was low (CV = 5.03%) indicating the great precision of the implemented trials [24, 41].

In addition, the coefficients estimates, standard errors and *P-*values of linear (A, B, C), quadratic (A^2^, B^2^, C^2^) and interaction (AB, AC, BC) terms of the regression model were presented in Table 3. The results showed that, the linear (A, B, C), interaction (BC) and quadratic (B^2^, C^2^) terms were significant, whereas the interaction (AB, AC) and quadratic (A^2^) terms were non-significant.

**Table 3:** Coefficient estimates, standard errors, and *P-*values of BBd

Term	Coefficient estimate	Standard error	*P*-value	95% CI Low	95% CI High	VIF
Intercept	175.27	6.95		157.40	193.13	
A-Enzyme units	76.30	4.26	< 0.0001	65.36	87.24	1.0000
B-Temperature	14.30	4.26	0.0201	3.36	25.24	1.0000
C-pH	-15.40	4.26	0.0152	-26.34	-4.46	1.0000
AB	0.6000	6.02	0.9245	-14.87	16.07	1.0000
AC	8.80	6.02	0.2035	-6.67	24.27	1.0000
BC	-41.60	6.02	0.0010	-57.07	-26.13	1.0000
A^2^	1.67	6.26	0.8008	-14.43	17.77	1.01
B^2^	46.47	6.26	0.0007	30.37	62.57	1.01
C^2^	71.87	6.26	< 0.0001	55.77	87.97	1.01

**Fig. 2:**
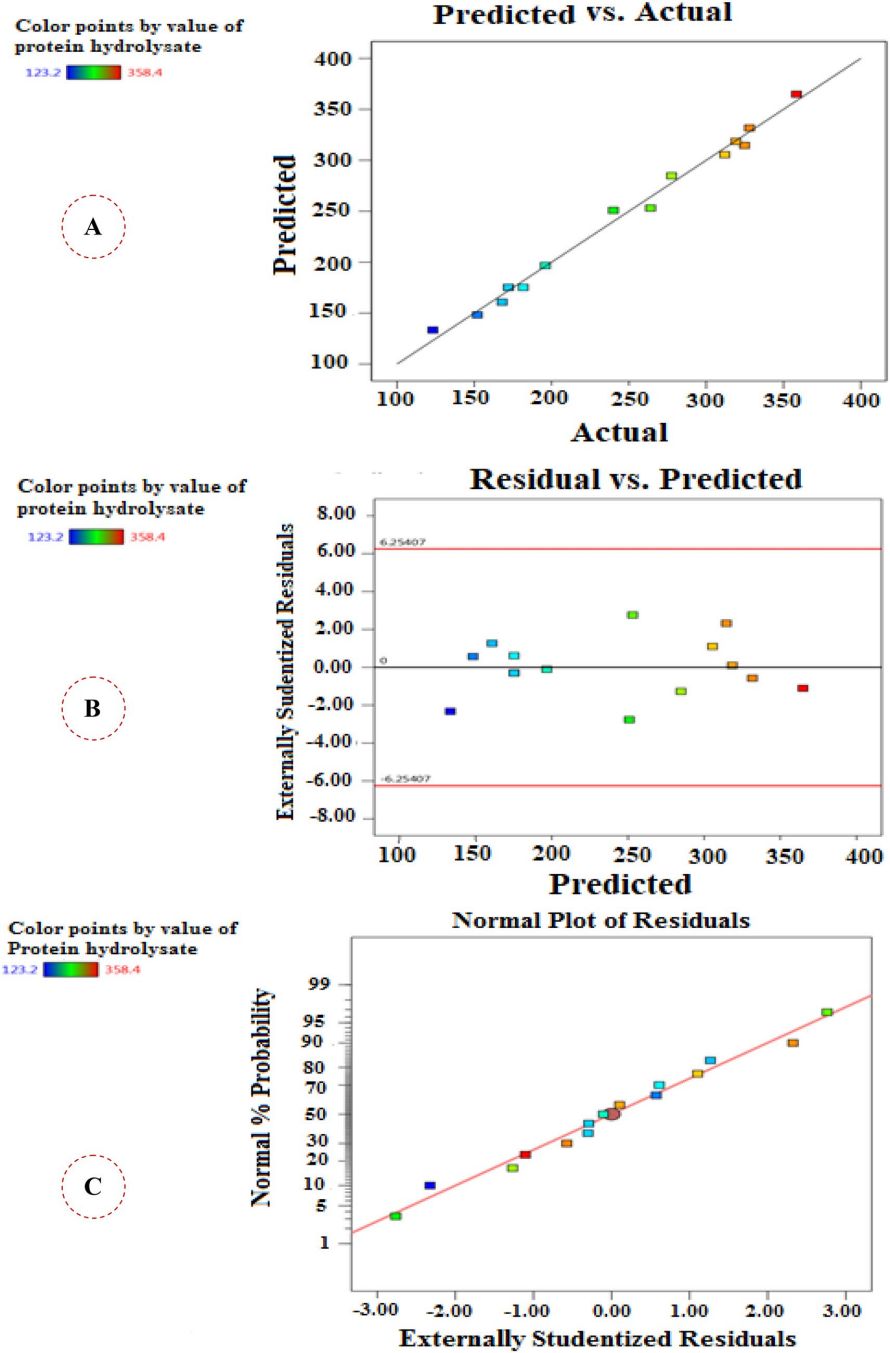
**(A)** Relationship between predicted and actual values **(B)** Plot of residuals against predicted values **(C)** Normal probability plot of residuals of BBd for protein hydrolysate optimization

The efficiency of the model was clearly emphasized through the high closure between the actual and predicted values of protein hydrolysate as shown in Fig. 2A. Likewise, the graph of residuals vs. predicted values (Fig. 2B) confirms the significance of BBd model due to the random dispersing of residuals around a horizontal zero reference. Moreover, the normal probability plot (Fig. 2C) showed that the residuals were closely outspread along a straight line which points to the accuracy of the experiments and the eligibility of the statistical model.

**Fig. 3:**
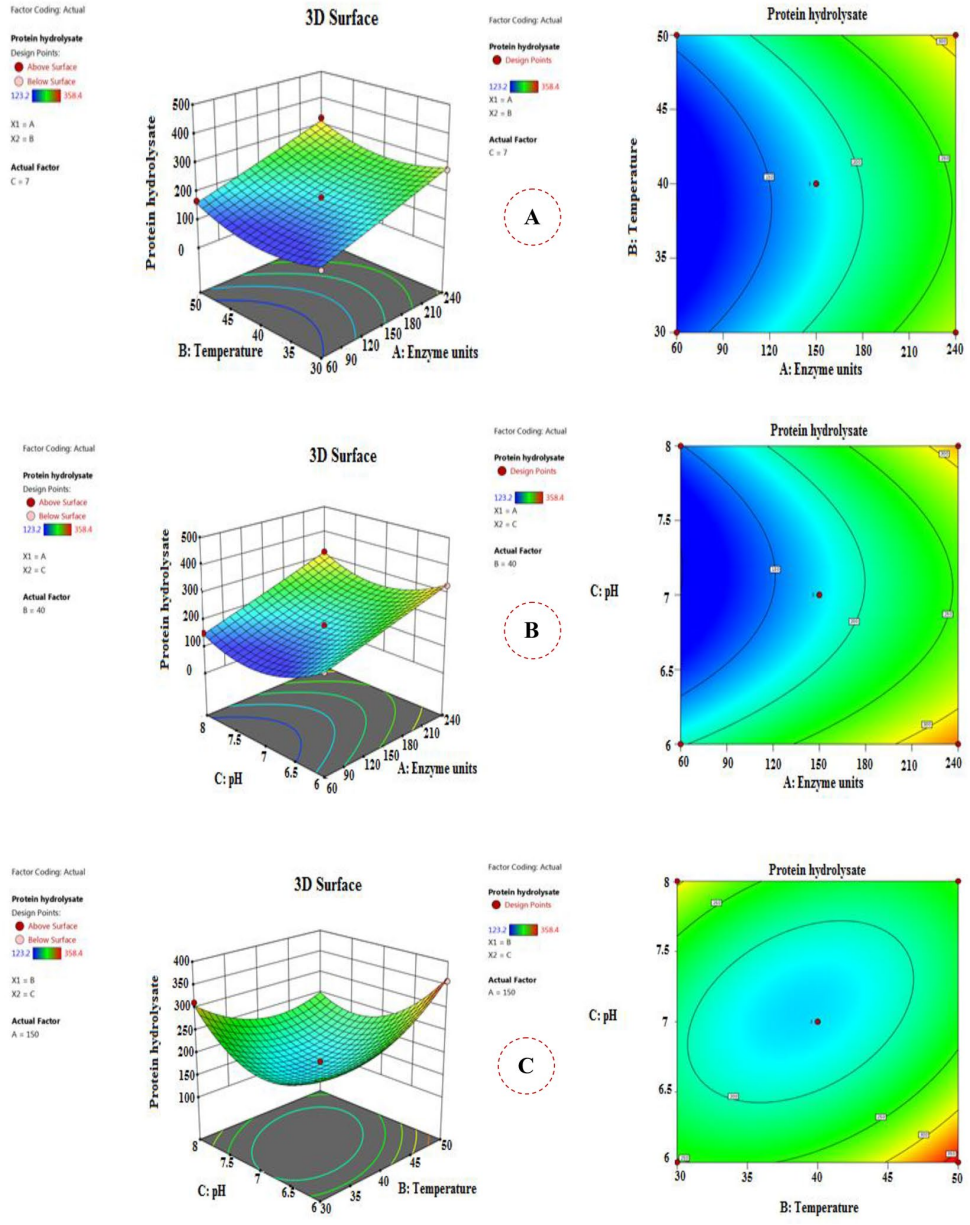
The 3D response surface and contour plots showing the interaction between each two variables affecting the protein hydrolysate yield **(A)** Enzyme units and Temperature **(B)** Enzyme units and pH **(C)** Temperature and pH

The response surface 3D graphs and contour plots of BBd offered the interactions among each 2 distinct variables whereas the other one was kept at its zero (central) level as displayed (Fig. 3). They provide a visional explanation for the variables interactions impact on protein hydrolysate production to obtain the best levels of the tested variables. Figure 3A illustrates the interaction between enzyme units and temperature while pH was maintained at its central level (7). The greatest protein hydrolysate yield was noted in trial 8 (358.4 mg/mL/g), which was recorded at the central level of enzyme units (150 U), high level of temperature (50 °C), and low level of pH (6.0). Additionally, Fig. 3B displays the interaction between enzyme units and pH while maintaining the temperature at its zero level (40 °C). In this case, the high levels of both enzyme units (240 U) and pH (8) achieved the maximum protein hydrolysate production (319.2 mg/mL/g). Besides, the influence of temperature and pH on protein hydrolysate yields while maintaining enzyme units at the central point (150 U) was observed in Fig. 3C.

### Validation and verification of BBd model

The validation of BBd model was confirmed by the performance of an experimental trial conducted at the optimum level of the tested variables (enzyme units 150 U, temperature 50 °C, and pH 6.0), which were predicted by the model and resulted in a maximum protein hydrolysate yield of 364.9 mg/mL/g. The experimental trial yielded a protein hydrolysate value of 362.5 mg/mL/g, which was very close to the predicted value (364.9 mg/mL/g) at approximately 99.3%, demonstrating a high precision degree of the model.

In this work, the application of BBd to optimize factors influencing protein hydrolysate yield from SC by *B. thuringiensis* protease showed a peak hydrolysate value of 362.5 mg/mL/g, which was 4.2-times higher than the initial conditions. Singh et al. [42] found that the ideal hydrolysis conditions for rice bran protein utilizing papain enzyme to achieve the maximum hydrolysate value via statistical CCD model were: enzyme/substrate ratio (0.035 w/w), temperature (50 °C), hydrolysis duration (150 min), and a pH (7.0).

Furthermore, optimizing the hydrolysis conditions of orange peel (OP 0.1 g, temperature 37.5 °C, and duration time 19.5 h) with *Aspergillus niger* WA 2017 protease raised the yield of protein hydrolysate by 5.3-times as reported by Ahmed et al. [8]. Moreover, the best conditions for protease to hydrolyze Yak whey protein concentrates using RSM optimization were: E/S ratio 7500 U/g, at 62 °C, pH 8.0, duration of 2.5 h [40]. It is clear that enzyme concentration plays a crucial role, and temperature significantly affects the enzymatic hydrolysis process [7, 15]. Finally, the optimized parameters for achieving the highest protein hydrolysate yield from SC using BBd with *B. thuringiensis* strain-MA8 protease were: 150 U enzyme units, 50 °C temperature, and pH 6.0 for duration of 24 h at 150 rpm. The data displayed in Table 4 show the comparison between unoptimized versus optimized conditions for protein hydrolysate yields from sesame cake (SC) using *B. thuringiensis* strain-MA8 protease.

**Table 4:** Comparison between un-optimized vs. optimized conditions of protein hydrolysate yields from Sesame cake (SC) using *B. thuringiensis* strain-MA8 protease

Protein hydrolysate conditions	Enzyme units (U)	Temperature (°C)	pH	yields mg/mL/g	Fold increase
Un-optimized	60	40	7.0	87.2	-
Optimized	150	50	6.0	362.5	4.2-fold

### Particle size of SH

**Fig. 4:**
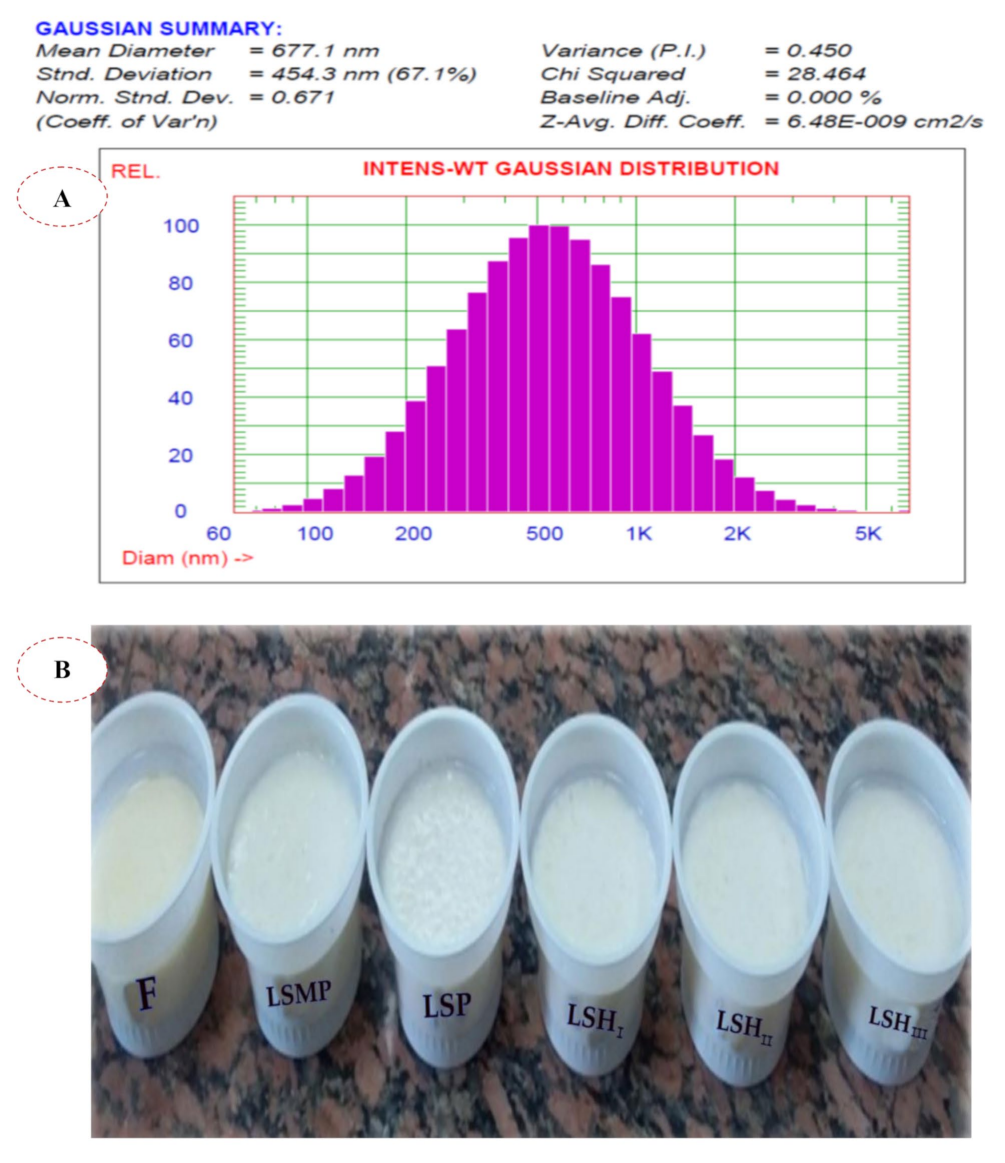
Particle size distribution of sesame cake protein hydrolysate **(A)** and Photo of yogurt samples with different protein sources **(B)** where: F (full-fat yogurt), LSMP (low-fat yogurt fortified with 2% skim milk powder), LSP (low-fat yogurt fortified with 2% sesame cake protein powder), LSH I, LSH II, LSH III (low-fat yogurt fortified with 1, 2, and 3% of Sesame cake protein hydrolysate, respectively)

As indicated in Fig. 4A, the enzymatic treatment of SC with *B. thuringiensis* strain-MA8 protease of SC produced major with a particle size greater than 2500 nm, primarily composed of polypeptides [43]. SH contained almost percentage of peptides with particle size ranging from 1277.30 to 2569.60 nm reached 90–99%, while 75–80% peptides with particle size ranging from 850.30 to 951.10 nm. Also, SH composed of peptides with particle size 541.10 nm (50%) and 344.70 nm (25%). Hence, the mean diameter of the particle size of SH using *B. thuringiensis* strain-MA8 protease is 677.10 nm. However, soy protein hydrolysate using Alcalase followed by heating at 90 °C for 20 min and homogenized at 8000 rpm for 6 min had a particle size of 7.1–9.3 μm which used as a fat replacer in ice cream processing [44]. Tanger et al. [45] demonstrated that the particle size ranged from 3 to 100 μm for pea protein microparticles and from 9 to 110 μm for potato protein microparticles; where pea and potato protein microparticles can be bigger than whey protein microparticles and still be perceived as creamy. The particle size threshold from which the particles are perceived as gritty is dependent on the hardness of the particle [46]. The particle size and the protein–protein interaction in the particles would render the created pea and potato protein a suitable fat replacer in theory [45].

### Yogurt production using SH

SH resulted from *B. thuringiensis* strain-MA8 protease treatment was employed to supplement of low-fat yogurt milk, in order to increase its total solids as well as improve the texture of the product when compared to FFY (F), LSMP, and LSP (Fig. 4B).

### Yogurt fermentation time

**Fig. 5:**
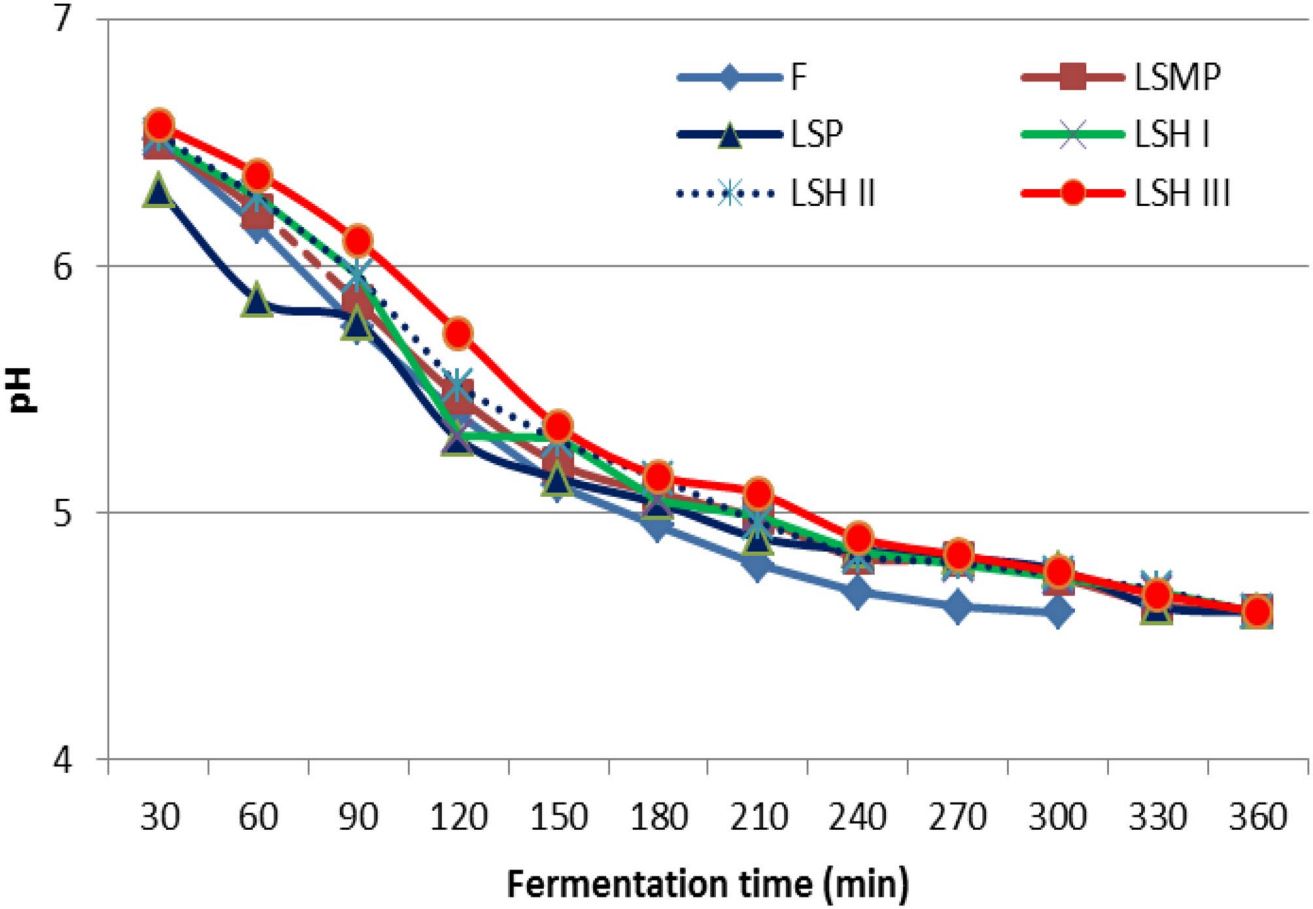
Fermentation curve of yogurt samples with different protein sources. Where: F (full-fat yogurt), LSMP (low-fat yogurt fortified with 2% skim milk powder), LSP (low-fat yogurt fortified with 2% sesame cake protein powder), LSH I, LSH II, LSH III (low-fat yogurt fortified with 1, 2, and 3% of Sesame cake protein hydrolysate, respectively)

The pH was noted to increase throughout the fermentation process until it reached 4.6, and the alterations in pH are presented in Fig. 5. The findings indicated that the fermentation duration of FFY was shorter compared to other yogurt treatments, whereas the incorporation of SH or SP powder prolonged the fermentation time of yogurt, nearing that of LSMP.

The addition of plant-derived components like moringa leaf powder, sea buckthorn mousse, and other anthocyanin-rich plants into the yogurt prolonged the fermentation time necessary to achieve a pH level between 4.5 and 4.6 [47]. Furthermore, Ma et al. [48] indicated that incorporating bovine and fish gelatin hydrolysate into yogurt milk may postpone yogurt fermentation, leading to reduced acid production (or slower acidification).

However, the reduction in yogurt milk pH during fermentation is caused by the effects of lactic acid production, which occurred due to LAB growth. In the present study, two strains of LAB were used including: *L. bulgaricus* and *S. thermophilus*. Throughout fermentation, those two strains grew together synergistically, *S. thermophilus* developed during the initial phase, reducing the mixture’s pH through free amino acids. This is due to the higher availability of peptides, which are necessary for the growth of *L. bulgaricus*. The growth of *L. bulgaricus* produced greater quantities of lactic acid, which significantly decreased the pH [49].

### Chemical characterization of yogurt

**Fig. 6:**
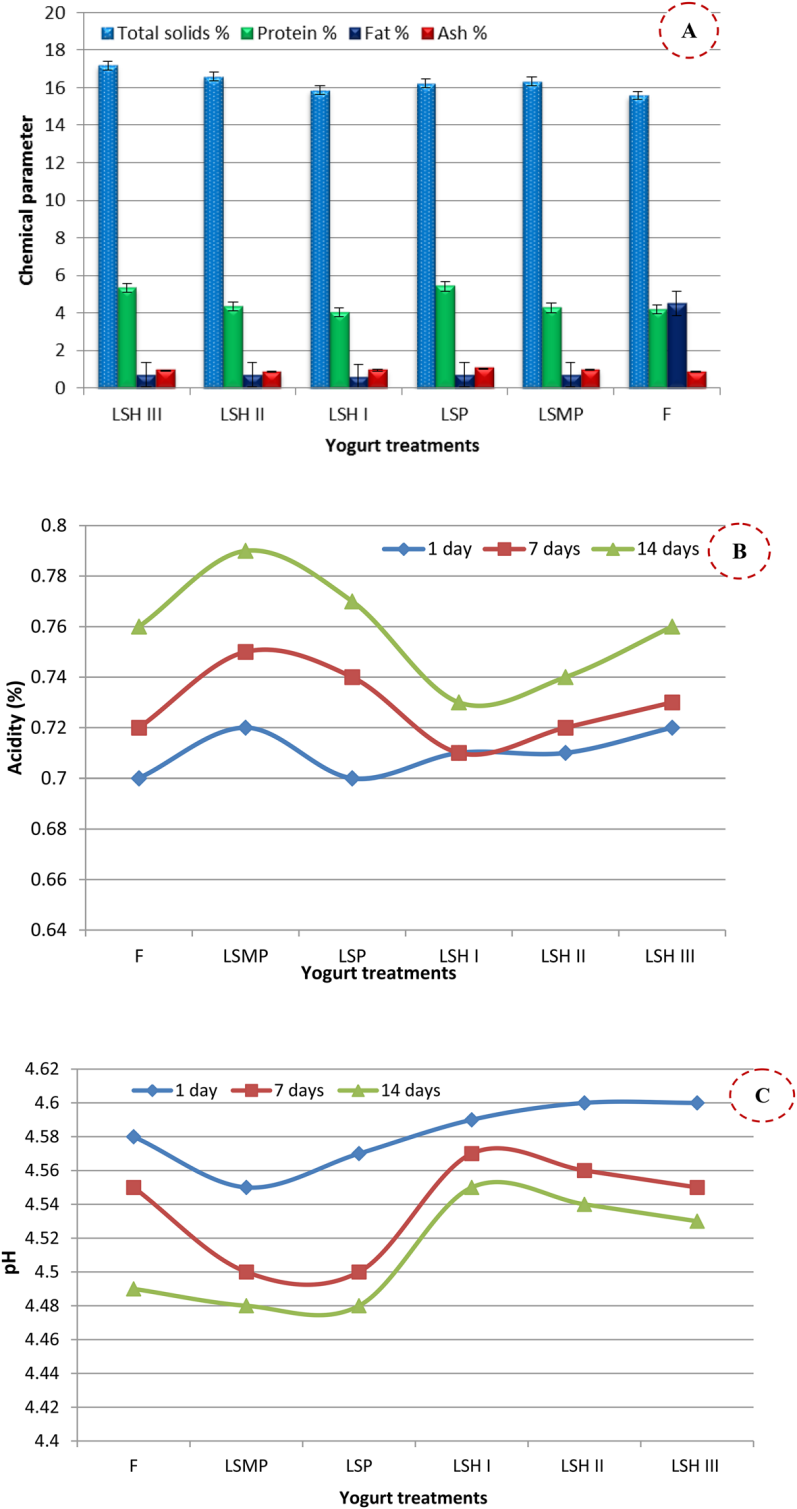
Chemical characterization **(A)**, Acidity **(B)**, and pH **(C)** of yogurt samples with different protein sources where: F (full-fat yogurt), LSMP (low-fat yogurt fortified with 2% skim milk powder), LSP (low-fat yogurt fortified with 2% sesame cake protein powder), LSH I, LSH II, LSH III (low-fat yogurt fortified with 1, 2, and 3% of Sesame cake protein hydrolysate, respectively)

Chemical characterization of yogurt revealed that there were no notable differences in total solids, protein, and fat levels among yogurt variations with various protein additions, such as skim milk powder and SP powder (Fig. 6A).

These findings were accordance with the report of Ma et al. [48]. Also, the total solids and protein levels in the LSH-yogurt samples (2, and 3%) were higher than FFY (Fig. 6A).

### Acidity of yogurt

The acidity levels of LSH-yogurt samples were close to FFY during storage for 15 days at 5 °C, whereas LSMP and LSP-yogurt samples exhibited a greater acidity level in comparison to FFY (Fig. 6B). However, in the opposite trend the yogurt pH results were presented in Fig. 6C. It could be noted that pH of LSMP and LSP was lower than that of FFY. No significant (*p* ≤ 0.05) differences in yogurt pH among FFY and LSH. Nonetheless, the yogurt pH of all samples decreased as the storage duration extended, attributed to increased lactic acid production possibly resulting from the synergistic LAB growth occurring during the storage period [46].

### Yogurt sensorial attributes

**Fig. 7:**
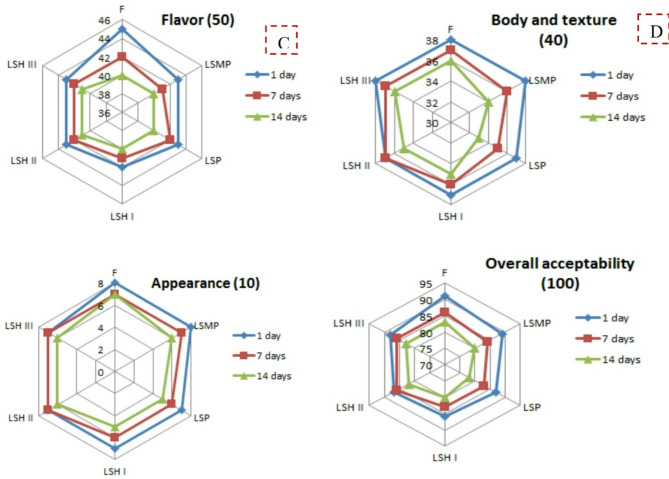
Sensorial attributes of yogurt samples with different protein sources. Flavor **(A)**, Body and texture **(B)**, Appearance **(C)**, and Overall acceptability **(D)**. Where: LSMP (low-fat yogurt fortified with 2% skim milk powder), LSP (low-fat yogurt fortified with 2% sesame cake protein powder), LSH I, LSH II, LSH III (low-fat yogurt fortified with 1, 2, and 3% of Sesame cake protein hydrolysate, respectively)

The sensorial evaluation of yogurt indicated that the flavor score of LSH-yogurt samples were comparable and nearly identical to FFY (Fig. 7A). The body and texture (Fig. 7B) and appearance (Fig. 7C) of the LSH III-yogurt sample (3%) resembled that of FFY, whereas the LSH II-yogurt sample (2%) was similar to LSMP. The overall acceptability findings indicated that LSH-yogurt samples (2 and 3%) were close to FFY over the 15-day storage period at 5 °C, without any appeared defects (Fig. 7D). Chatterjee et al. [50] noted that the flavor, color, and appearance scores of yogurt samples showed tolerance and palate acceptance due to the addition of tryptic whey protein hydrolysate for all levels.

### Amino acids profile of yogurt

The amino acid profile of yogurt samples showed that total essential amino acid content (total EAA) of the chosen LSH II-yogurt sample based on the sensorial attributes in Fig. 7 was nearly similar to that of the control yogurt (Table 5). Also, the results showed that the total amino acids content (total AA) of LSH II-yogurt sample was similar to that of the control (F) yogurt, while the total AA of LSP-yogurt sample was close to low fat yogurt with SMP. Hence, the total EAA/total AA ratio of LSH II-yogurt, which used 2% Sesame cake protein hydrolysate resulted from *B. thuringiensis* strain-MA8 protease treatment, was close to control yogurt samples.

**Table 5:** Amino acids profile of yogurt samples with different protein sources

Amino acids (mg/g protein)	Yogurt treatments			
	F (full-fat yogurt)	LSMP (low-fat yogurt with 2% skim milk )	LSP (low-fat yogurt with 2% sesame cake protein)	LSH II (low-fat yogurt with 2% sesame cake protein hydrolysate)
Aspartic acid	113.86	78.53	126.68	90.84
Glutamic acid	77.64	83.29	91.07	94.91
Serine	36.82	45.28	39.25	41.57
Glycine	20.58	21.58	22.44	16.83
Histidine	33.00	52.38	40.71	44.77
Arginine	92.61	60.18	64.04	72.93
Threonine	59.96	55.60	65.82	53.54
Alanine	43.43	47.13	42.22	45.34
Proline	21.95	27.06	27.66	26.85
Tyrosine	45.22	42.98	66.16	50.05
Valine	51.32	44.69	59.12	59.22
Methionine	44.08	43.55	72.84	58.74
Cysteine	24.14	29.85	28.16	34.38
Isoleucine	47.67	71.36	62.23	72.25
Leucine	62.53	67.47	72.03	51.23
Phenylalnine	80.05	89.02	53.96	68.35
Lysine	95.02	94.60	21.29	68.48
Total EAA	506.28	523.25	446.22	495.97
Total AA	949.88	954.55	955.68	950.28
EAA/TAA ratio	0.533	0.548	0.467	0.522

### Yogurt textural profile

**Fig. 8:**
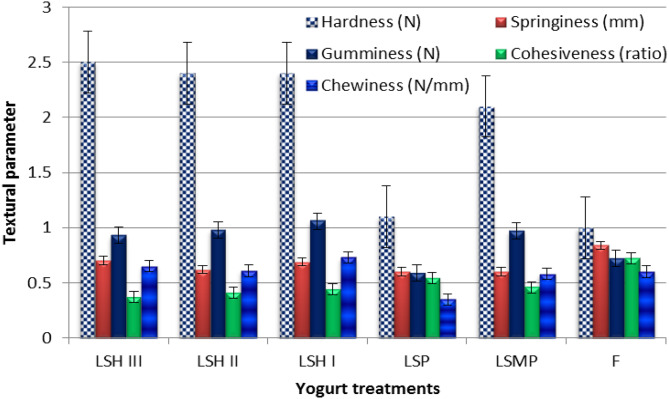
Textural profile of yogurt samples with different protein sources. Where: F (full-fat yogurt), LSMP (low-fat yogurt fortified with 2% skim milk powder), LSP (low-fat yogurt fortified with 2% sesame cake protein powder), LSH I, LSH II, LSH III (low-fat yogurt fortified with 1, 2, and 3% of Sesame cake protein hydrolysate, respectively)

It is evident from Fig. 8 that the texture profile of the yogurt samples produced validated the sensorial attributes, indicating that the hardness of yogurt treatments with varying protein additives (SMP, SP, and SH) was greater than of FFY; while the hardness is increased gradually with SH level increased which affects the sensorial attributes of yogurt. Textural and overall acceptability scores were increased with SH level increased and then decreased in the highest level of SH. Additionally, the springiness, gumminess, and cohesiveness of LSH-yogurt samples were similar to LSMP. It could be attributed to the interaction of the amino acids from the added proteins and milk casein, which stabilizes the protein network in the resulting yogurt during both the fermentation and storage processes [51, 52], which confirms the improvement of the body and textural scores of the resulted yogurt samples with SH. Therefore, the characteristics of SP could similarly affect network formation, such as hydrophobicity, foaming capacity, turbidity characteristics, and stability. This phenomenon led to an improvement in the viscosity and consistency of the yogurt [53]. Moreover, the increased LSH-yogurt hardness values indicate an excellent texture and appearance in the final yogurt characteristics. Nonetheless, the inclusion of casein hydrolysate with alcalase in the yogurt sample consistently resulted in greater hardness compared to the control yogurt sample [48]. Additionally, a negative correlation exists between yogurt hardness and syneresis susceptibility [54]. Also, in the process of protein network formation, LAB released protons through hydrolytic action, thereby neutralizing the negatively charged protein complexes. The formed structure was stabilized through Van der Waals forces, hydrogen bonds, and hydrophobic interactions [55]. However, the presence of protein during yogurt milk fermentation impacted the structural formation in the final yogurt product, thereby altering its physical characteristics [56, 57]. Furthermore, increased levels of protein aided in the production of acid whey, which hardened the yogurt structure [57].

### Nutritional value of yogurt

**Fig. 9:**
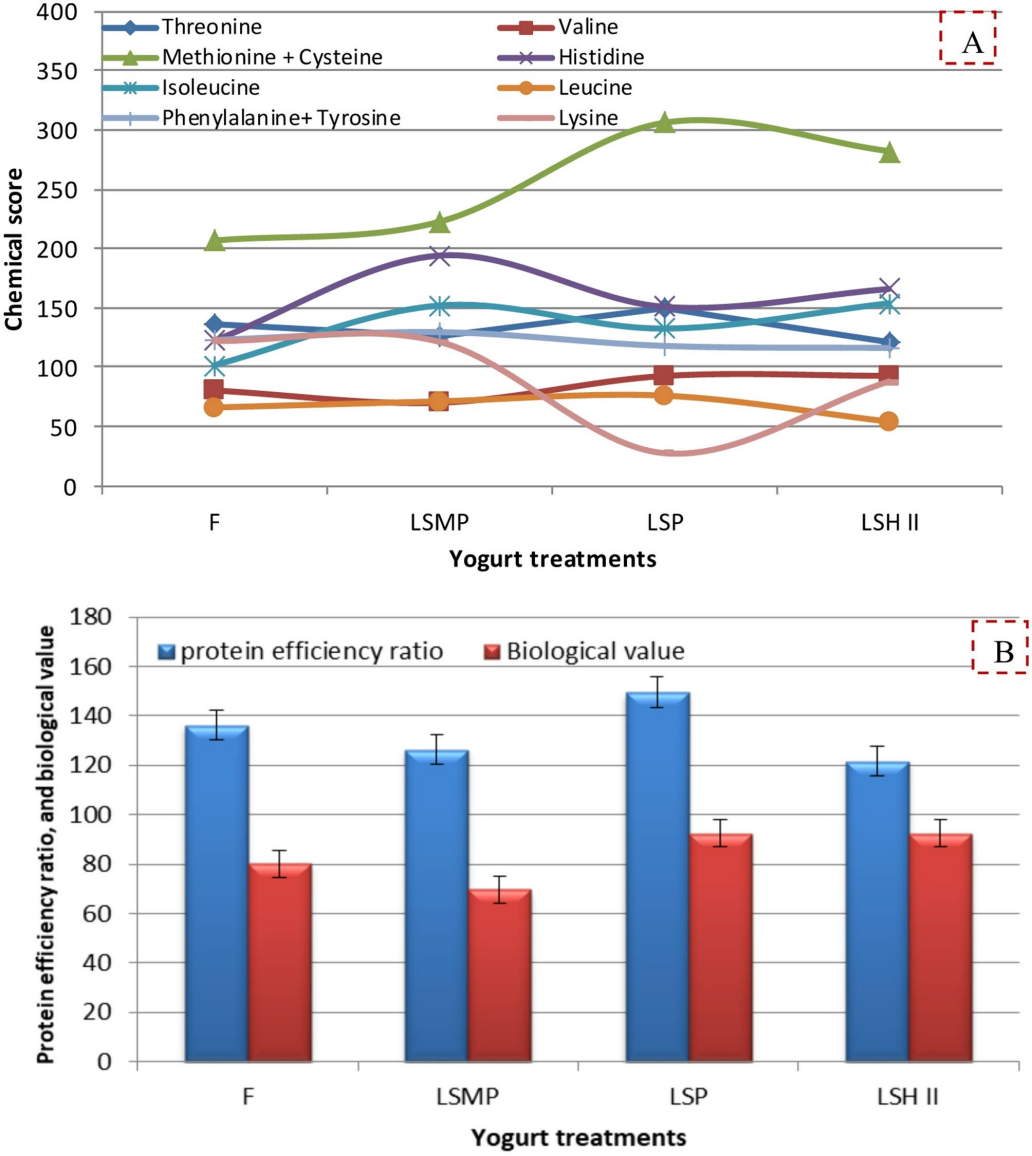
**(A)** Chemical score and **(B)** protein efficiency ratio and biological value of yogurt samples with different protein sources. Where: F (full-fat yogurt), LSMP (low-fat yogurt fortified with 2% skim milk powder), LSP (low-fat yogurt fortified with 2% sesame cake protein powder), LSH I, LSH II, LSH III (low-fat yogurt fortified with 1, 2, and 3% of Sesame cake protein hydrolysate, respectively)

Chemical score is a comparison of the amount of the limiting amino acid in a food with the amount of that same amino acid in a reference food (milk was used as standardized protein in the present study); while Protein efficiency ratio determines the effectiveness of protein in foodstuffs. Biological value measures the proportion of absorbed nitrogen which is retained and presumably utilized for protein synthesis and therefore reflects true protein quality. The concept of BV has the merit that it can be used to assess requirements of protein derived from foods with known quality differences, because BV is directly related to the efficiency of protein utilization. In the present study such parameters of protein quality in yogurt were calculated based on their amino acids content [55]. Hence, the nutritional value of yogurt indicated that the addition of either SP powder or SH (the selected LSH II-yogurt sample based on sensorial attributes) enhanced the calculated chemical score of certain amino acids (e.g., valine, methionine + cysteine, and isoleucine) as presented in Fig. 9A. Also, the calculated protein efficiency ratio and the biological value (Fig. 9B) of LSP–yogurt were similar to that of the control yogurt.

## Conclusion

Sesame cake, a by-product high in protein, was hydrolyzed with the use of *Bacillus thuringiensis* strain-MA8 protease. Improved hydrolysis conditions through RSM using Box-Behnken design increased the hydrolysate yield by 4.2 times, achieving 362.5 mg protein/g of Sesame cake. The incorporation of Sesame cake protein hydrolysate (SH) as a fat replacer in low-fat yogurt production led to a slight delay in acid production, enhanced nutritional value and texture, and improved stability of low-fat yogurt, bringing its characteristics closer to those of full-fat yogurt. Interestingly, throughout a 14-day storage period at 5 °C for low-fat yogurt enriched with SH, no defects were detected. Nevertheless, the protein hydrolysate from Sesame cake could be a beneficial component for producing low-fat yogurt. The Sesame cake protein hydrolysate can be used in other food products, which is our goal in future studies.

## Electronic supplementary material

Below is the link to the electronic supplementary material.

Supplementary Material 1

Supplementary Material 2

Supplementary Material 3

## Data Availability

No datasets were generated or analysed during the current study.
