# Supplementary material for: Bioactive protein hydrolysate from Sesamum indicum L. residue as a novel fat substitute by protease: production optimization and application in low-fat yogurt production
Source: Microb Cell Fact. 2025 May 27;24:123. doi: 10.1186/s12934-025-02748-3 (PMC12107946; doi:10.1186/s12934-025-02748-3)
Supplement: Supplementary file 1 — Supplementary Material 1 [file 12934_2025_2748_MOESM1_ESM.pdf]

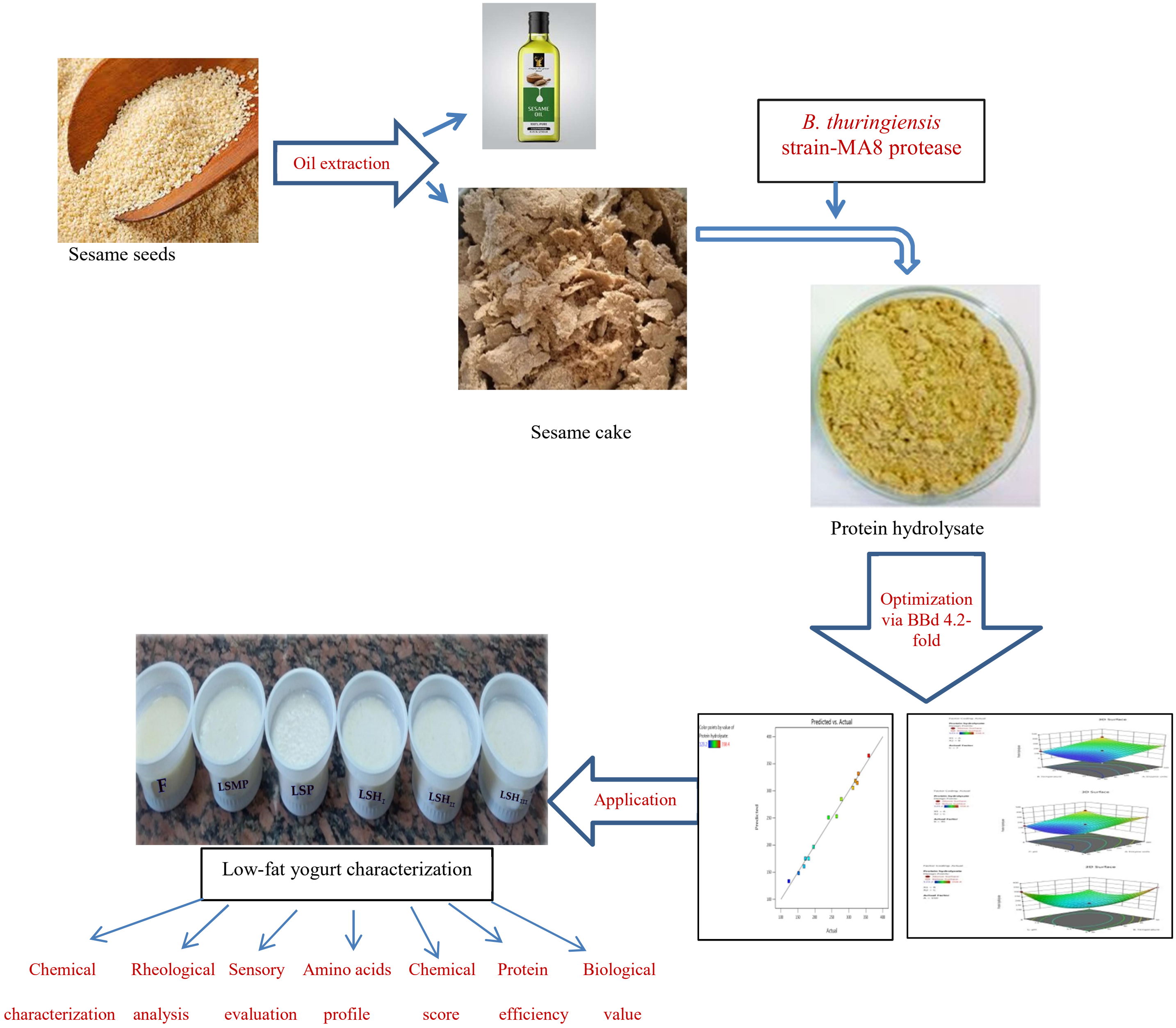
Sesame seeds
Oil extraction
Sesame cake
B. thuringiensis strain-MA8 protease
Protein hydrolysate
Optimization via BBd 4.2-fold
Application
F
LSMP
LSP
Low-fat yogurt characterization
Chemical characterization
Rheological analysis
Sensory evaluation
Amino acids profile
Chemical score
Protein efficiency
Biological value
