# Supplementary material for: Bioactive protein hydrolysate from Sesamum indicum L. residue as a novel fat substitute by protease: production optimization and application in low-fat yogurt production
Source: Microb Cell Fact. 2025 May 27;24:123. doi: 10.1186/s12934-025-02748-3 (PMC12107946; doi:10.1186/s12934-025-02748-3)
Supplement: Supplementary file 2 — Supplementary Material 2 [file 12934_2025_2748_MOESM2_ESM.doc]

**Highlights**

- Eco-friendly way to convert Sesame cake residues to bioactive protein hydrolysate (SH).
- Box-Behnken optimization promote hydrolysate yield by 4.2-fold.
- Low-fat yogurt supplemented with SH takes a shorter fermentation time.
- SH-yogurt acceptance scores were close to full fat yogurt (FFY) with no defects.
- Total solids, protein contents, and amino acids of SH-yogurt were higher than FFY.
