# Supplementary material for: Bioactive protein hydrolysate from Sesamum indicum L. residue as a novel fat substitute by protease: production optimization and application in low-fat yogurt production
Source: Microb Cell Fact. 2025 May 27;24:123. doi: 10.1186/s12934-025-02748-3 (PMC12107946; doi:10.1186/s12934-025-02748-3)
Supplement: Supplementary file 3 — Supplementary Material 3 [file 12934_2025_2748_MOESM3_ESM.doc]

All authors agree to publish in the Microbial Cell Factories
